# Associations between perfluorinated chemicals and serum biochemical markers and performance status in uremic patients under hemodialysis

**DOI:** 10.1371/journal.pone.0200271

**Published:** 2018-07-17

**Authors:** Wen-Sheng Liu, Yen-Ting Lai, Hsiang-Lin Chan, Szu-Yuan Li, Chih-Ching Lin, Chih-Kuang Liu, Han-Hsing Tsou, Tsung-Yun Liu

**Affiliations:** 1 Division of Nephrology, Department of Medicine, Taipei City Hospital, Zhongxing Branch, Taipei, Taiwan; 2 School of Medicine, National Yang-Ming University, Taipei, Taiwan; 3 College of Science and Engineering, Fu Jen Catholic University, New Taipei City, Taiwan; 4 Institute of Environmental and Occupational Health Sciences, School of Medicine, National Yang-Ming University, Taipei, Taiwan; 5 Department of Physical Medicine and Rehabilitation, National Taiwan University Hospital Hsin-Chu Branch, Hsinchu, Taiwan; 6 Department of Nursing, Yuanpei University, Hsinchu, Taiwan; 7 Department of Child Psychiatry, Chang Gung Memorial Hospital and University, Taoyuan, Taiwan; 8 Division of Nephrology, and Department of Medicine, Taipei Veterans General Hospital, Taipei, Taiwan; 9 College of Medicine & Graduate Institute of Business Administration, Fu Jen Catholic University, New Taipei City, Taiwan; 10 Institute of Food Safety and Health Risk Assessment, National Yang-Ming University, Taipei, Taiwan; University of Cincinnati, United States

## Abstract

Perfluorooctanesulfonate (PFOS) and perfluorooctanoic acid (PFOA) are commonly used perfluorinated chemicals (PFCs). PFCs are mainly excreted by urine. Uremic patients tend to accumulate toxins in their body and have poor functional status. We investigated the associations between PFCs and the clinical profile of uremic patients under hemodialysis (HD). Liquid chromatography tandem mass spectrometry coupled with isotope dilution was used to quantify PFOA and PFOS. We enrolled 126 patients under regular HD. Compared with previous research, the concentration of PFOA was lower, but that of PFOS was higher in uremic patients than in the general population. The levels of PFOA and PFOS in uremic patients before dialysis were 0.52 (ng/ml) and 21.84 (ng/ml) respectively. The PFOA level remained unchanged but that of PFOS decreased to1.85 ng/mL after dialysis. PFOS can be removed by HD. Patients using hypertensive medication had a lower PFOS then those who did not. The PFOS level was negatively correlated with the duration of the HD session and patient performance status, but positively correlated with levels of cholesterol, chloride (an indicator of acidemia), ferritin, and total protein. (p<0.05). The association with serum protein may explain the long half-life of PFCs in humans. This is the first study which investigated PFCs in uremic patients and showed PFCs are associated with adverse effects in this population.

## Introduction

Perfluorinated chemicals (PFCs) are used as surfactants in various industries and consumer products because of their unique properties as repellents of dirt, water and oils. The most well-known and widely used PFCs are perfluorooctanoic acid (PFOA), perfluorooctane sulfonic acid (PFOS) and their derivatives belonging to the group of perfluoroalkylated substances. These two PFCs comprise the majority of all PFCs and are potential toxic endocrine disruptors. In addition, these two PFCs have a high persistence in the environment. [1] Bioaccumulateion and biomagnification of these substances occur in the food chain and ecosystem through exposure and intake [2]. Humans can absorb PFCs through oral intake or inhalation. PFCs are hard to eliminate due to enterohepatic cycling [3]. In daily life, drinking water, air dust, food wrapping material and nonstick pans are all potential exposure routes for PFCs [4]. PFOA and PFOS are stable materials and resist photodegradation [5, 6]. Their half-lives after hydrolysis are longer than six months in the human body [7].

In animal studies, PFCs has been correlated with cancer, abnormal neural behavior, and relapsing health problems [1, 8]. Human studies also proved that intrauterine exposure can result in low birthweights and adverse birth outcomes [9, 10]. Further evidence showed that exposure to PFCs is related to adverse effects such as decreased sperm count, glucose disequilibrium, metabolic syndrome, attention deficit hyperactivity disorder, and abnormal thyroid function [11–13].

As a result, PFCs have gradually been banned due to their toxicity. In May, 2000, the 3M Company abandoned the use of PFOS. By 2005, Sweden through the Stockholm Convention declared PFOS a “persistent organic pollutant”. In December, 2006, the European Union banned marketing and usage of PFOS, followed by Canada in January 2007. The European Union suspected that PFOA posed a similar threat. The 3M Company discontinued production of PFOA in 2003. In 2007, the U.S. Environmental Protection Agency came to agreement with factories on stepwise decreases in the manufacturing and use of PFOA with discontinuation of all PFOA by 2015 [14]. However, many other developing countries are still producing these PFCs.

PFCs are mainly eliminated by the kidney[15]. The membrane slit of a glomerulus is about 25–60 nm while the diameters of PFOA and PFOS are larger about 300 nm) [16]. PFCs are secreted from serum to urine by organic transporters on the renal tubular cells, not simple filtration from the glomerulus. Toxic substances may accumulate in uremic patients due to damage to renal tubular cells [17]. One study also showed elevated PFC levels are associated with chronic kidney disease (CKD) [18].

The prevalence of CKD and renal failure are high in Taiwan [19, 20]. Uremic patients tend to accumulate toxins in their body and commonly suffer from cardiovascular diseases (CVD) and poor function status. There are several biochemical markers (such as cholesterol, albumin and hemoglobin) related to CVD and functional status [21, 22]. However, the PFC level in uremic patients under hemodialysis (HD) has not been investigated previously and the influence of dialysis on the PFC level has not been reported. We hypothesize that PFC levels may be higher in uremic patients and might change after dialysis. Higher PFC levels may be associated with markers of CVD and poor performance scores.

We used liquid chromatography tandem mass spectrometry (LC-MS/MS) with isotope dilution to quantify the serum concentration of PFOA and PFOS in uremic patients under HD and compared the difference before and after HD. Then we analyzed the association between PFC and patient characteristics, HD treatment and biochemical profiles. We tried to verify whether PFCs can be removed by HD and the association of PFOA and PFOS with the clinical profiles of uremic patients.

## Materials and methods

### Ethics statement

The study was approved by the Institutional Review Board /Ethics Committee of Taipei Veterans General Hospital before the trial began. Participants gave written informed consent in accordance with the Declaration of Helsinki.

### Inclusion and exclusion criteria

This is a cross-sectional study at a medical teaching hospital in northern Taiwan. We enrolled patients over 18 years old who had received maintenance HD therapy three times weekly for at least 3 months. Patients who had peritoneal dialysis or transplantations were excluded. Patients who had blood transfusions or intravenous lipid nutrition supplements, propofol, dopamine, chemotherapy, antibiotics, or immunosuppressants (such as streoids or cyclosporine) were also excluded. A total of 126 uremic patients completed the study. We checked the concentration of PFOA and PFOS in the serum of these patients before and after dialysis.

#### Data collection

Personal and clinical data such as age, gender, body weight, cause of renal failure (diabetes or chronic glomerulonephritis), medications related to uremia (hypertension medication, iron supplements, vitamin D usage and erythropoietin) and hemodialysis treatment profile (duration of each HD session, loading and maintenance doses of anticoagulants blood flow and dialysate flow, potassium and calcium concentrations of the dialysate, artificial kidney surface area, urea reduction ratio, Kt/V, clearance) were obtained from the medical records. Laboratory biochemical profiles were gathered at the beginning of the month, prior to hemodialysis. Activities of daily living (ADL) were evaluated by a physician using the Karnofsky performance status (range from 0 [death] to 100 [fully normal functioning])

LC-MS/MS coupled with isotope dilution was used to simultaneously quantify PFOA and PFOS in the serum. The differences in PFOA and PFOS levels in the serum before and after dialysis were evaluated. Blood samples were collected at a teaching hospital in northern Taiwan.

#### Measurement of PFOA and PFOS

Serum samples were stored at −80°C before analysis. The analytical method for PFOA and PFOS was as follows: First, the frozen serum samples were thawed at 4°C and then vortex mixed for 30 seconds to homogeneity. A 50 μL serum sample in a polypropylene centrifuge tube was vortexed with 50 μL of 1% formic acid (pH 2.4) for 30 seconds. Then 40 μL of acetonitrile and 1μL of 10 μg/mL internal standard solution (^13^C_4_-PFOA and ^13^C_4_-PFOS, Wellington Laboratories Inc., Guelph, Ontario, Canada) were added to each sample before the second vortex. The sample was sonicated for 20 min and then centrifuged at 18,000×g for 20 min. The supernatant was collected and then filtered through a 0.22 μm polyether sulfone syringe filter into a screw cap vial.

**Fig 1:**
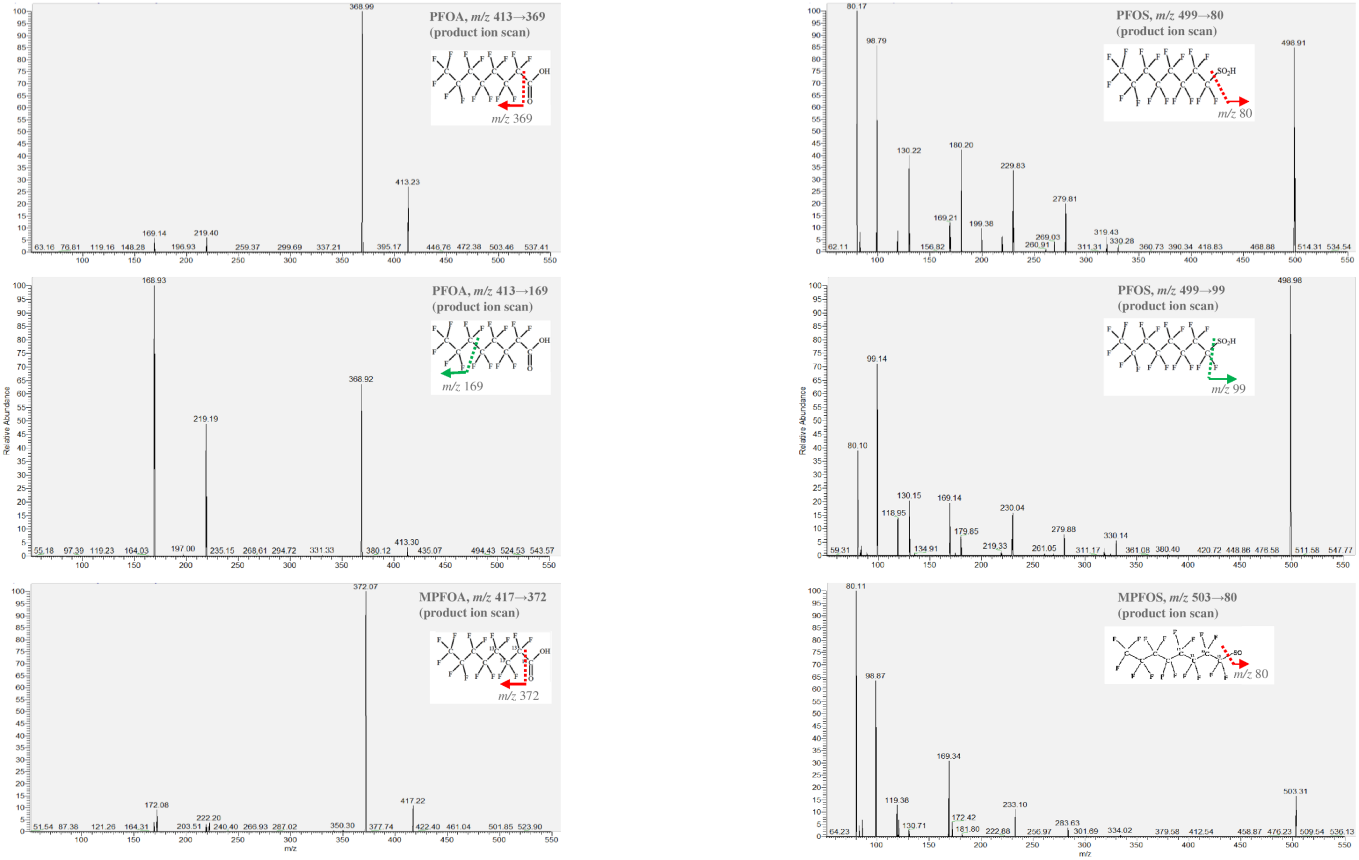
Product ion scan of perfluorooctanoic acid (PFOA) and perfluorooctane sulfonate (PFOS).

The LC-MS/MS system used in this study comprised an Agilent 1100 series system (Agilent Tech., Santa Clara, CA, USA) coupled to a Finnigan TSQ Quantum Discovery Max spectrometer system (Thermo Electron Corporation, Breda, Netherlands) with an electron spray ionization source in negative ion mode. LC-MS/MS was carried out with isotope dilution for simultaneous quantification of PFOA and PFOS in the serum. A sample (5 μL) was injected onto a 2.0 mm × 150 mm Capcell Pak 3 μm C18 column (Shiseido Co., Tokyo, Japan) with mobile phases consisting of 10 mM ammonium acetate in water (A) and pure acetonitrile (B) delivered at a constant flow rate of 0.2 mL/min. The mobile phase was kept at 30% B for 3 min after injection. A gradient was applied in 3 min to 65% B, then in 5 min to 100% B where it was kept for 7 min. The column was then conditioned at 30% B for 1.5 min. Optimized mass spectrometry parameters were as follows: spray ion voltage 3,000 V, capillary temperature 210°C, sheath gas pressure 10 arbitrary units, auxiliary gas pressure 5 arbitrary units, ion sweep gas pressure 4 arbitrary units, collision gas pressure 1.0 mTorr, and dwell time 100 msec. The detection was carried out in selective reaction monitoring (SRM) mode. The collision energy (V) and SRM transitions monitored were as follows: 10 V, m/z 413→369 for PFOA; 12 V, m/z 417→372 for ^13^C_4_-PFOA; 40 V, m/z 499→80 for PFOS; 40V, m/z 503→80 for ^13^C_4_-PFOS. (Fig 1) We used LC-MS/MS to quantify PFOA (*m/z* 413→369), PFOS1 (*m/z* 499→80) and PFOS2 (*m/z* 499→99) in the serum. Because the concentration of PFOS1 (*m/z* 499→80) was the higher than PFOS2, we used it as the main particle for PFOS for further analysis.

### Statistical analysis

The sample size of uremic patients was determined based on an effect size to detect significance of the intervention effect of HD on the change of concentration of PFCs. If we permitted a 5% chance of type I error (α = 0.05), with a power of 90%, assuming the difference before and after t HD is at least half of the standard derivation of each parameter, then approximately 84 uremic patients would be required to have a sufficient sample size. Sera from126 uremic patients were collected in this study. (Table 1)

**Table 1:** Comparison of serum PFC concentrations in uremic patients (n = 126) before and after HD.

t-test	pre-HD	post -HD	pre/post
	mean±SD	mean±SD	paired t-test
Men (%)	73(57.9%)		
Age (years)	59.75±14.79		
PFOA (ng/mL)	0.52±0.27	0.50±0.29	.707
PFOS (ng/mL)	21.84±48.12	1.85±0.91	.001*

*p<0.05 (pre/post, before vs. after HD)

HD: hemodialysis, PFC: perfluorinated chemicals; PFOA: perfluorooctanoic acid; PFOS: perfluorooctanesulfonate.

The concentrations of PFOA and PFOS before and after HD were analyzed by paired- t test. Comparisons of different subgroups of the uremic patients such as those with different comorbidities (diabetes mellitus [DM] chronic glomerulonephritis), or under different medical treatments (hypertension treatment, iron supplementation, use of vitamin D, erythropoiesis stimulating agents were analyzed by independent t-test) (Table 2)

**Table 2:** Comparisons of patient data and medication profiles with PFOA and PFOS levels in different subgroups of uremic patients using the independent t-test (n = 126).

Pre HD	Yes	PFOA (Yes vs. No)				t-test	PFOS (Yes vs. No)				t-test
(n = 126)	(%)	mean	±SD	mean	±SD	p	mean	±SD	mean	±SD	p
Age>45 (vs. age< = 45)	83.3	0.56	0.28	0.38	0.15	.010*	10.23	13.20	24.01	52.47	.185
Age>50(vs. age< = 50)	75.4	0.57	0.29	0.40	0.17	.003*	24.80	54.56	12.17	17.68	.326
Men (%) (vs. women)	57.9	0.49	0.29	0.57	0.25	.204	20.61	41.76	23.04	56.21	.805
DM (%)(vs. non-DM)	41.0	0.60	0.33	0.48	0.21	.064^+^	16.58	24.21	25.31	59.93	.438
CGN(%)(vs. non-CGN)	26.2	0.52	0.24	0.53	0.29	.805	25.96	54.16	19.34	46.24	.584
Medication(vs. without)											
anti-HTN	20.0	0.51	0.29	0.52	0.27	.811	2.53	3.87	27.04	53.61	.001*
Oral Fe supplement	62.6	0.51	0.27	0.53	0.28	.767	11.93	14.53	33.19	67.72	.073^+^
Oral Vit.D treatment	12.0	0.50	0.23	0.52	0.28	.762	10.40	17.54	23.74	51.61	.446
ESA treatment	96.0	0.52	0.27	0.78	0.05	.179	22.14	48.90	4.57	4.80	.615

*p<0.05,

^+^p<0.10

DM, diabetes mellitus; CGN, chronic glomerular nephritis; HTN: hypertension; ESA, erythropoiesis stimulating agent; PFOA, perfluorooctanoic acid; PFOS, perfluorooctanesulfonate.

The associations of the clinical parameters of patient characteristics, hemodialysis treatment, hemogram, and biochemical profiles to the PFOA and PFOS levels in the serum were analyzed by linear regression. (Tables 3 & 4) Data statistical analysis was done using SPSS statistical software (version 19.0; IBM, Armonk, New York, USA). Distributions of continuous variables in groups were expressed as mean ± SD and compared by Student’s t-test. A v *p* value less than 0.05 was considered statistically significant.

**Table 3:** Relationship of age and dialysis profiles of to serum concentrations of PFOA and PFOS in uremic patients using linear regression (n = 126).

Pre HD	mean	±SD	PFOA β	ng/mL p	PFOS β	ng/mL p
**PFOA**	**0.53**	**0.27**			**.000**	**.993**
**PFOS**	**21.68**	**48.34**	**.000**	**.993**		
Age (year)	59.75	14.79	.001	.675	.354	.320
HD duration(month)	59.75	67.75	3.2e-6	.345	-.002	.576
EPO dosage/month	20,898	10,167	.000	.283	.001	.392
Karnofsky performance status	76.12	16.97	-.002	.213	-.858	.008*
Blood flow (ml/min)	272.18	33.36	.000	.718	-.048	.773
Dialysate flow(ml/min)	510.15	56.99	.000	.491	-.043	.581
HD frequency(/week)	2.98	0.12	.005	.675	.NA	. NA
dialysis time(hours)	4.023	0.37	-.077	.281	-27.528	.029*
AK surface size(m^2^)	1.90	0.27	-.077	.500	-19.340	.348
AC Initial dose (U)	1319.77	981.76	-7.2e-5	.008*	-.008	.056^+^
AC maintainance dose (U)	219.89	272.46	-4.3e-5	.737	.018	.413
[Ca] in dialysate(mEq/L)	3.02	0.27	-.045	.718	-2.433	.911
[K] in dialysate(mEq/L)	2.00	0.43	-.724	.190	-31.227	.751
Urea reduction ratio	0.74	0.05	.175	.757	-11.456	.910
Kt/V (Gotch)	1.39	0.22	.049	.714	-6.366	.788
Kt/V ((Daugirdes))	1.64	0.29	.033	.763	-11.388	.555
Ccr (ml/min)	6.03	2.00	.001	.970	2.040	.495
nPCR	1.02	0.26	-.042	.761	-15.172	.539
TAC urea	37.20	10.20	-.002	.502	-.501	.425

* p<0.05,

^+^ p<0.10

DM, diabetes mellitus; CGN, chronic glomerulonephritis; HD, hemodialysis; AK, artificial kidney; AC, anticoagulant; Ccr, creatinine clearance; nPCR, normalized protein catabolic rate; TAC urea, time average concentration for urea; PFOA, perfluorooctanoic acid; PFOS, perfluorooctanesulfonate; EPO, erythropoietin.

**Table 4:** Relationship of hemogram and biochemical profiles to serum concentrations of PFOA and PFOS in uremic patients using linear regression.

Pre HD (n = 126)	mean	±SD	PFOA β	ng/mL p	PFOS β	ng/mL p
WBC (x10^3^/ul)	6.83	2.46	-.021	.195	2.088	.479
RBC (x10^6^/ul)	3.36	0.50	-.025	.711	-7.929	.501
Hb (g/dl)	9.89	1.20	-.003	.905	2.168	.648
Hct (%)	30.39	3.67	-.006	.498	.580	.710
MCV (fl)	91.17	7.23	.000	.947	2.168	.023*
Platelet (x1000/ul)	195.71	68.31	-.001	.147	-.067	.441
Cholesterol (mg/dl)	154.70	35.57	.001	.497	.358	.011*
Triglycerides (mg/dl)	136.62	78.87	.000	.422	.011	.853
Glucose (mg/dl)	136.81	56.86	.000	.511	.036	.697
Total protein (gm/dl)	6.94	3.98	.016	.714	21.522	.005*
Albumin (gm/dl)	3.92	0.37	.049	.542	9.024	.526
Globulin	2.95	4.02	.038	.152	8.236	.076^+^
AST (IU/L)	22.70	10.45	.006	.069^+^	-.481	.423
ALT (IU/L)	18.77	10.88	.005	.167	-.548	.363
Alk-P (IU/L)	93.72	83.06	.000	.856	-.020	.709
Total Bilirubin (mg/dl)	0.54	0.15	.201	.458	-70.672	.139
Uric acid (mg/dl)	6.99	1.18	.027	.301	-8.001	.085^+^
Sodium (mEq/l)	138.92	2.73	.001	.959	-1.064	.587
Potassium (mEq/l)	4.56	0.66	-.033	.510	1.769	.842
Cl (mEq/l)	98.83	5.62	.010	.077^+^	2.529	.013*
Calcium (mg/dl)	9.27	0.86	.040	.250	15.034	.012*
Phosphorus (mg/dl)	4.69	1.33	-.038	.103	-2.653	.525
Creatinine (mg/dl)	9.18	2.22	-.014	.285	-2.458	.285
BUN (mg/dl)	61.69	17.09	-.001	.628	.327	.683
Fe (μg/dl)	59.39	22.15	-.001	.481	.055	.827
UIBC (μg/dl)	188.99	55.09	.000	.827	-.184	.093^+^
TIBC (μg/dl)	248.38	48.10	.000	.923	-.211	.080^+^
Ferritin(ng/ml)	488.95	422.60	.000	.794	.072	2.6e-9*
TSAT (%)	24.78	9.93	-.001	.773	.777	.186
Al (ng/ml)	15.16	9.85	-.006	.173	-.080	.561
iPTH (pg/ml)	205.26	301.44	.000	.543	-0.16	.315

* p<0.05,

^+^ p<0.10

(WBC, white blood cells; RBC, red blood cells; Hb, hemoglobulin; Hct, hematocrit; MCV, mean corpuscular volume; AST, aspartate aminotransferase; ALT, alanine transaminase; Alk-P, alkaline phosphatase; UIBC, unbound-iron binding capacity; TIBC, total iron-binding capacity; TSAT, transferrin saturation; PFOA, perfluorooctanoic acid; PFOS, perfluorooctanesulfonate; BUN, blood urea nitrogen; Al, aluminum; iPTH, intact parathyroid hormone.

## Results

**Fig 2:**
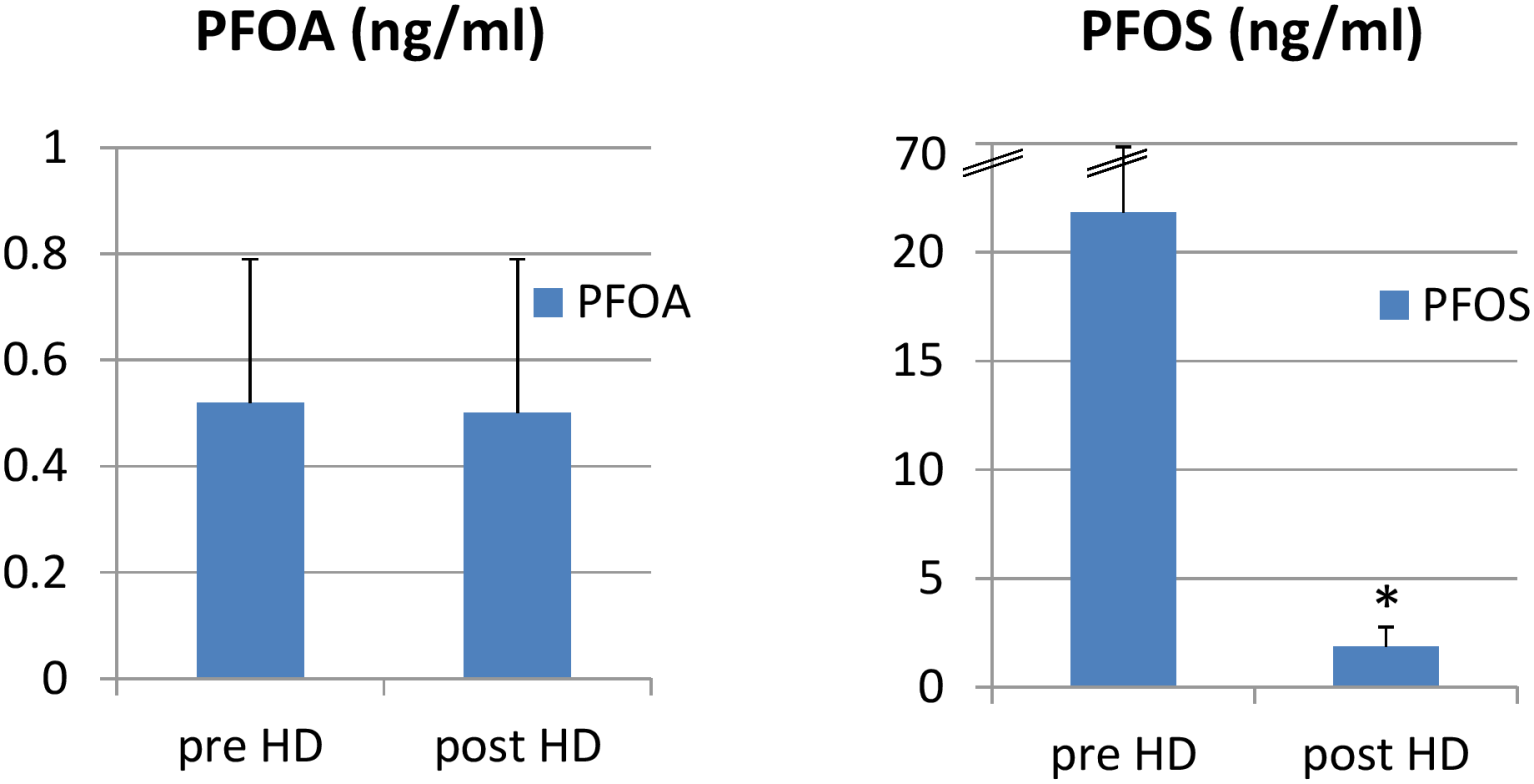
Comparison of PFOA and PFOS concentrations in uremic patients (n = 126) before (pre HD) and after HD (post HD) (ng/ml) (p = 0.707 for PFOA, p = 0.001 for PFOS) (* compared with pre-HD value with paired t–test, p<0.05).

The average age of the uremic patients in this study was 59.75 years (SD = 14.79) and 58% were men. The average concentrations of PFOA and PFOS in uremic serum were 0.52 (ng/ml) (SD = 0.27 ng/ml), and 21.84 (ng/ml) (SD = 48.12 ng/ml) before HD. The average concentration of PFOA remained the same (0.50 ng/mL, SD = 0.29) and that of PFOS decreased to 1.85 ng/mL (SD = 0.91) ng/mL after HD. PFOS can be removed by dialysis. (Table 1 and Fig 2)

**Fig 3:**
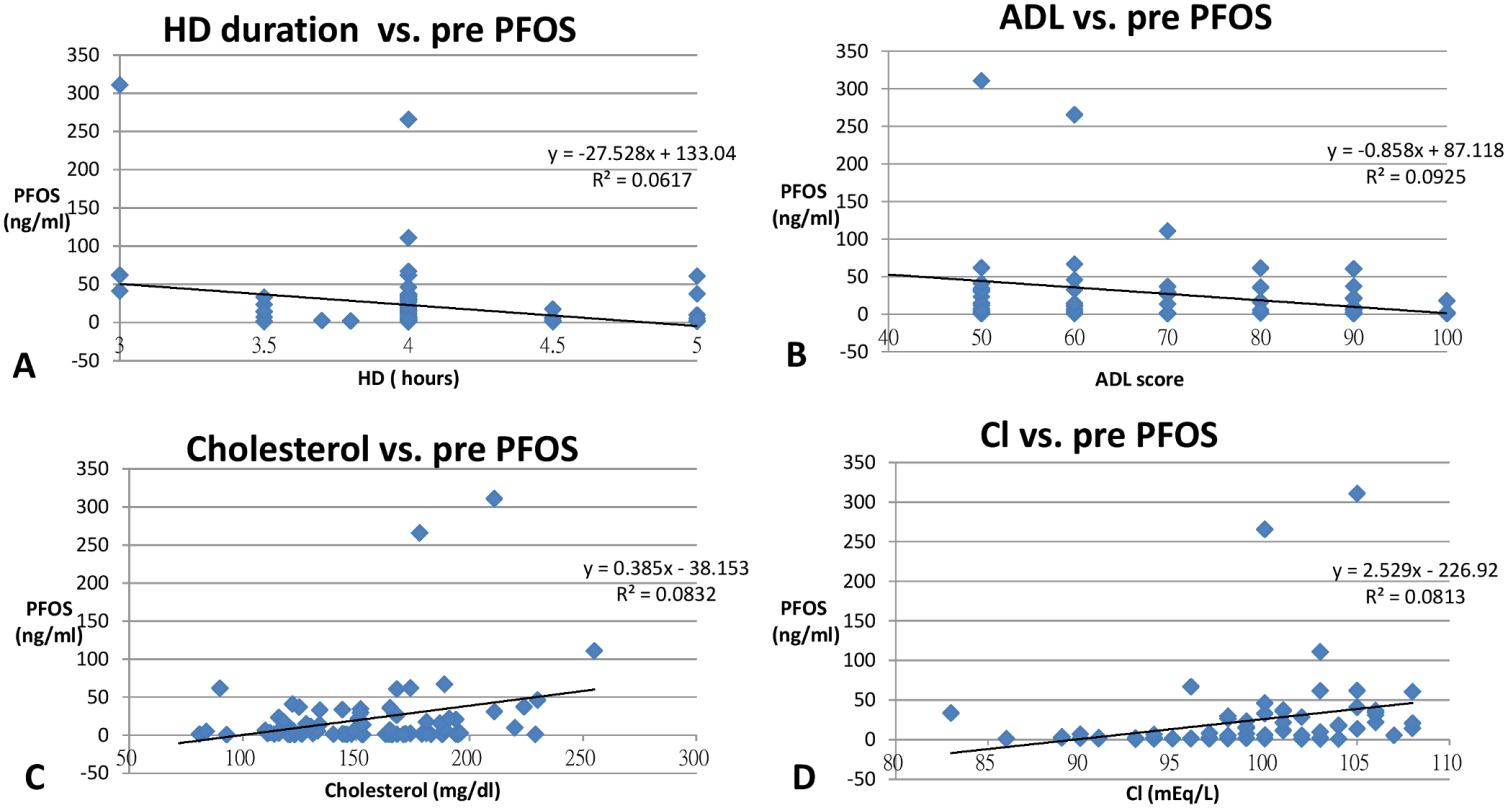
Linear regression of hemodialysis (HD) duration (hours) (p = 0.029) (A), Karnofsky performance status score for activities of daily living (ADL) (p = 0.008) (B), serum cholesterol (mg/dl) (p = 0.011) (C) and chloride (Cl) (mEq/L) (p = 0.013) (D) (X-axis) with serum PFOS concentration (ng/ml) (Y-axis) (all p <0.05).

**Fig 4:**
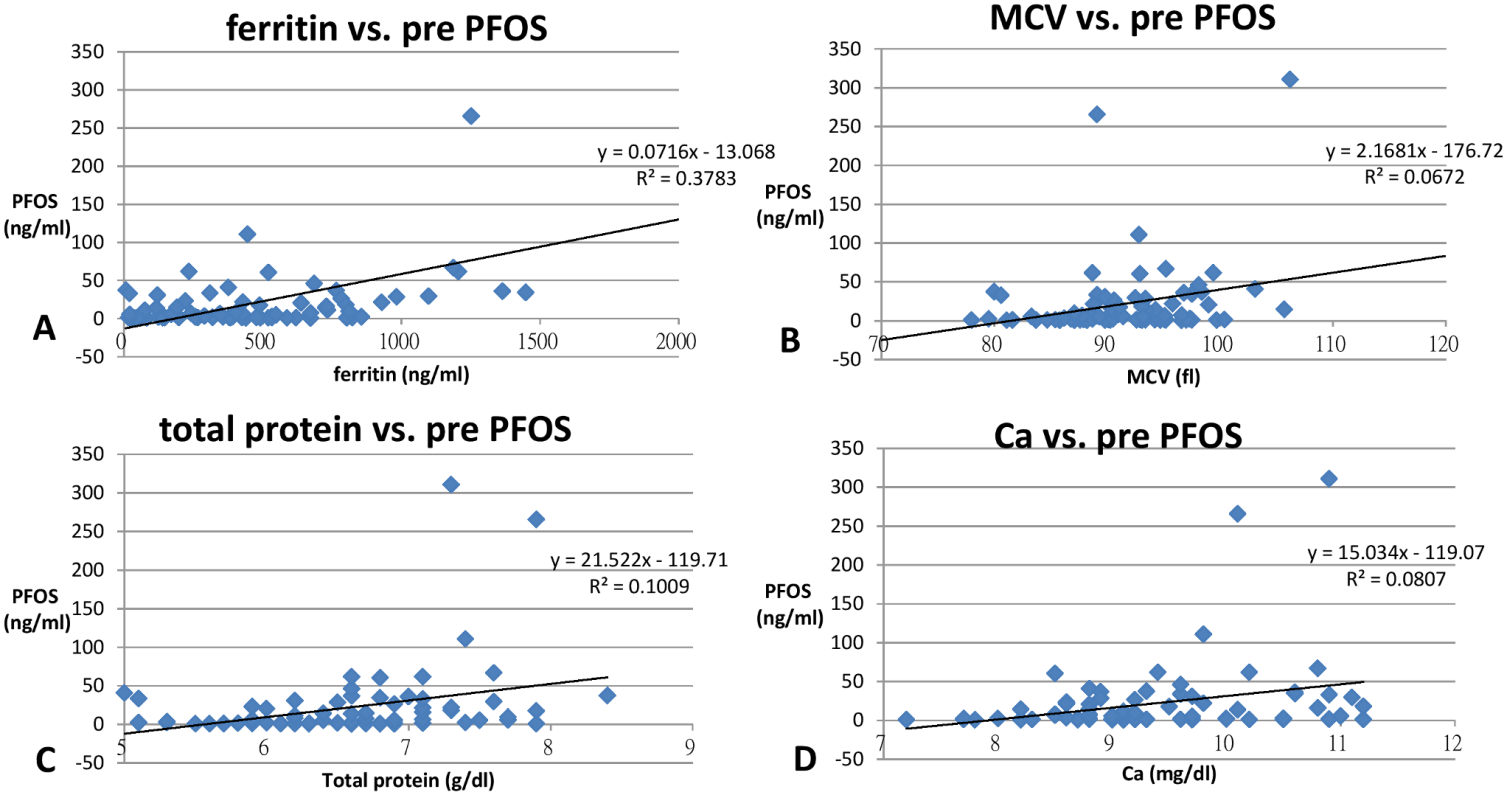
Linear regression of ferritin (ng/ml) (p<0.001) (A), mean corpuscular volume (MCV) (fl) (p = 0.023) (B), protein (g/dl) (p = 0.005) (C) and Ca (mg/dl) (p = 0.012) (D) (X axis) with serum PFOS concentration (ng/ml) (Y axis) (all p <0.05).

The concentrations of PFOA and PFOS in different subgroups were analyzed by independent t-test and are shown in Table 2. The correlation of patient characteristics, hemodialysis treatment, hemogram, and biochemical profiles to PFOA and PFOS were analyzed by linear regression and are shown in Tables 3 and 4. All significant associations between PFCs and clinical parameters (patient characteristics, treatment and biochemical profiles) are discussed below and are shown in Figs 3 and 4.

## Discussion

In previous studies in Taiwan, the serum concentration of PFOA was 3.22 ng/ml and that of PFOS was 8.52 ng/ml in healthy people, which are similar to levels in other countries. (PFOA: 1.5–10 ng/mL, PFOS: 5.0–35 ng/mL) [23]. In Korea, the concentration of PFOA was 2.85 ng/ml and PFOS was 10.23 ng/ml in the general population[24]. Our study showed lower PFOA levels (mean± SD: 0.52 ± 0.27 ng/ml) but higher PFOS levels (21.84 ± 48.12 ng/ml) in uremic patients than the above-mentioned studies. (Fig 2)

The PFOA concentration is lower in the serum of uremic patients than in the general population. (Table 1) (Fig 2) A possible explanation is that today people encounter more products using PFOA in their living environment than they did formerly. Uremic patients are older than the general population. A study in 2014 in Taiwan showed children had higher serum concentrations of PFCs than adults and the concentration showed an increasing trend compared with results from 2006 and 2008 [25]. However, more studies are needed to provide evidence to support this point.

In subgroup analysis, PFOA levels were higher in older uremic patients than younger patients (age less than 45 years vs. older; and age less than 50 years vs. older) (Table 2) This implies that PFOA accumulates in the human body and is hard to eliminate [26]. Comparison of uremic sera before and after dialysis showed that PFOA can not be removed by HD. (Table 1) Therefore, older patients accumulate more PFOA than younger ones due to longer exposure.

Although PFOA can not be removed by HD, there was a negative association with the intial loading dose of heparin and the PFC concentration. (for PFOA, p = 0.008; for PFOS, p = 0.056) Previous study showed perfluorinated chemicals are not found intracellularly within the red blood cells, thus the concentration of PFCs in serum is higher than that in whole blood [7]. One possible explanation is that the loading heparin before each HD session may affect the volume of serum extracted from the blood and therefore decrease the PFC concentration in the serum.

Patients with DM had higher (although not significantly) PFOA levels than those without DM. (p = 0.064) (Table 2). A study in 2009 also showed that PFOA is associated with metabolic syndrome and hyperglycemia in adolescents and adults. [12] In assciation analysis of biomarkers with PFOA, we found a postive (although not significant) linear assciation with aspartate aminotransferase (p = 0.067) (Table 4). This may imply that PFOA poses a harmful effect on the liver which controls glucose homeostasis [27].

Patients taking antihypertensives had a protective effect from PFOS accumulation compared with other patients (p<0.001) (Table 2). This might be related to the renal protective effect of antihypertensive medication and more PFC elimination by urine [28]. Uremic patients may still have residual renal function and urine output. More than forty percent of uremia in Taiwan is a result of DM [20]. More than half of the hypertension medications used in the study group were renin-angiotensin-aldersterone system (RAAS) blockers. This may be partially explained by the renal protection of RAAS blockers in uremic patients with DM -related CKD [29].

Uremic patients have much higher concentrations of PFOS than the general population. PFCs are mainly excreted by urine. PFOS can be removed by dialysis as PFOS significantly decreased after each dialysis. (Fig 2) Linear regression also showed a negative correlation of serum PFOS with number of hours of dialysis in each HD session. (p = 0.029) (Fig 3A).

PFOS was highly negatively correlated to the Karnofsky performance status score (p = 0.008) but postively correlated to cholesterol (p = 0.011) (Fig 3B & 3C) This finding is compatible with previous studies showing that PFOS is correlated with CVD [30]. Higher serum concentrations of PFOS have been associated with an increase in the thickness of the carotid intimal media (a marker of CVD) even in adolescents and young adults [26]. Previous study showed high serum cholesterol may induce CVD and dementia and affect performance status [31]. Thus PFOS may be associated with a poor prognosis in uremic patients as the Karnosfsky performance status score is a powerful predictor of patient survival [32].

We also found PFOS was significantly positively associated with chloride (Cl^-^), an indicator of acidemia (p = 0.013) (Fig 3D). The mechanism of acidemia may involve the organic anion exhanger located in the renal tubules. PFOS is an anion and can be excreted by absorbing another anion, such as chloride [33]. Our finding is compatible with a previous study showing PFOS has negative impact on bone mineral density (BMD) as acidemia contributes to lower BMD [34].

Our study indicates PFOS may interact with serum protein, which may explain the long half life of PFC in serum. (Fig 4) PFOS is a negatively charged anion and interacts with positively charged proteins such as ferritin [35]. This explains the positive association of PFOS and ferritin (p<0.001). Ferritin is also an indicator of iron storage involving erythropoiesis and is postively correlated with the size of the RBC, the mean corpuscular volume (MCV). [22] So PFOS is also postively correlated with the MCV (Fig 4A & 4B) The correlation of PFOS and total protein (p = 0.005) may also be explained by the interaction PFCs and serum protein [36]. As serum calcium is bound to albumin (one major component of serum protein) [37], Ca is highly correlated with PFOS as well. (p = 0.049) (Fig 4C & 4D) However, in multivariant linear regression for the above mentioned four variables (ferritin, MCV, total protein, Ca), only ferritin and total protein remained independent factors (p<0.05) which is compatible with our explanation.

PFOS was not positively correlated with uric acid in our study (p = 0.085), which was not compatible with a previous finding that PFOS induces hyperuricemia [38]. A possible explanation is that our patients are under dialysis and uric acid can be removed through dialysis. So uric acid may not show a positive correlation with PFOS in HD patients.

In summary, the concentrations of PFC were related to the age of the patients and may be influenced by their dialysis or treatement (medication for hypertension, loading dose of heparin or hours of dialysis). PFOA may be associated with worsening liver function and glucose metabolism. However, a limitation of this study is that our current sample size may not have been big enough to detect minor influences of PFCs on humans. Among the other biochemical parameters of uremic patients, cholesterol and choloride are highly correlated to the PFOS concentration. PFOS is also associated with poor patient performance status, which may imply a poor prognosis. Furthermore, PFOS may interact with serum protein and ferritin, which may contribute to the long half life of PFC in the human body.

## Conclusion

This is the first study that fully investigated PFCs in uremic patients and reported that one PFC (PFOS) can be removed by HD. PFCs may be associated with CVD in this population as PFOS is highly correlated with cholesterol and inversely correlated with patient performance status. Further study is needed to investigate the causal relationships. We hope our study can draw more attention to environmental toxins in uremic patients and the general population.
